# Evolutionary features of thyroid cancer in patients with thyroidectomies from 2008 to 2013 in China

**DOI:** 10.1038/srep28414

**Published:** 2016-06-22

**Authors:** Xiaoyun Liu, Lijun Zhu, Zhixiao Wang, Dai Cui, Huanhuan Chen, Yu Duan, Meiping Shen, Hui Lu, Zhihong Zhang, Jiawei Chen, Erik K. Alexander, Tao Yang, Xiaodong Wang

**Affiliations:** 1 Department of Endocrinology, The First Affiliated Hospital of Nanjing Medical University, Nanjing, China; 2 Department of Children’s Health care, Nanjing Maternity and Child Health Care Hospital Affiliated to Nanjing Medical University, China; 3 Department of Surgery, The First Affiliated Hospital of Nanjing Medical University, Nanjing, China; 4 Department of Pathology, The First Affiliated Hospital of Nanjing Medical University, Nanjing, China; 5 Thyroid Unit, Division of Endocrinology, Metabolism and Diabetes, Department of Medicine, Brigham & Women’s Hospital and Harvard Medical School, Boston, USA

## Abstract

To evaluate the characteristics of thyroid carcinoma over time, we carried out a retrospective study to illustrate the evolutionary features of thyroid carcinoma. All records of thyroidectomies from the First Affiliated Hospital of Nanjing Medical University from 2008 to 2013 were obtained focusing on pathological diagnosis, size, local lymph node metastasis (LNM) of the tumors. The thyroid cancer detection rate increased from 24.6% to 41.5% significantly (*P* < 0.05). Papillary thyroid carcinoma (PTC) remained to be the most common type counting 86.4% of all thyroid carcinomas. In all 1,704 PTCs, microPTC (mPTC) with maximum diameter less than or equal to 10 mm has become the dominant form taking up 56.5% of all PTCs in 2013 while only 43.1% in 2008. The mean maximum tumor size has decreased from 17.8 mm to 12.2 mm significantly (*P* < 0.05). However, the average age, female dominance, and local LNM remained similarly in the past six years. Logistic regression test showed that the determinants for local LNM were age, gender and tumor size. mPTC has become the most common form of thyroid carcinoma detected during thyroidectomies in China while other features of thyroid carcinoma remained similarly in the recent years.

Thyroid cancer is the most common endocrine cancer^1^. The incidence of thyroid cancer has increased dramatically worldwide, especially in Asian countries including China^2^,^3^,^4^,^5^,^6^,^7^. Thyroid cancer has become the most common form of cancer among Chinese women aged below 30 years old^6^. Our previous study has showed that by using ultrasound the prevalence of thyroid nodule was 46.6% in population aged above 40 years old^8^. However, only 7–15% of them are supposed to be cancerous according to the recent guideline from American Thyroid Association (ATA)^5^. Due to the high prevalence of this clinic scenario, selection of nodules with the highest risks of malignancy for thyroidectomy would be more reasonable^9^ and cost-effective^10^. These modalities include clinical evaluation, thyroid function test, ultrasound, FNA and molecular test if possible^1^,^9^,^11^. Although there are currently several guidelines to walk us through the pathway of diagnosing and treating patients with thyroid nodules, some major discrepancies exist among guidelines from different regions and organizations^1^,^5^,^12^,^13^,^14^. The cut-off size for performing FNA is one of them. The ATA guideline does not recommend performing FNA routinely for nodules less than 1 cm, however the guidelines from AACE/ETA and Korean Society of Radiology do not hold the cut-off strictly^13^,^14^. One the other hand, in real practice a recent study showed that the practitioners generally held a lower threshold for FNA than the cut-off recommended by ATA^15^. With more surveillance modality being applied for screening for thyroid cancer^2^,^4^,^16^,^17^,^18^, it is reasonable to speculate the size of thyroid cancer might decrease annually. However, it is more intriguing to know whether the nature of these tumors remains the same or not as marked by for example the local lymph node metastasis (LNM) one of common parameter for cancer progression specially for those less than 1 cm. Due to that uncertainty, the primary objective of current study was to evaluate the evolutionary features of thyroid carcinoma, especially for the size and the lymph node metastasis.

## Materials and Methods

Data were collected from Electronic Record Registration System in the Record Room and Pathological Diagnosis Registration and Reporting system in the Department of Pathology both from the First Affiliated Hospital of Nanjing Medical University, one of the largest academic hospitals of eastern China from 2008 to 2013 when both electronic systems were available since 2008. We reviewed all thyroid pathological diagnosis reports. Clinical data were also reviewed by inquiring the admission number in the Electronic Medical Record system when first available since 2009. All the data were obtained by chart review at a Record Room by X.L., L.Z., D.C., Z.W., H.C. and Y.D. We excluded data without precise documentation for the size of the tumor and those with invalid year. For patients with multiple entries in the same year and/or different years, we only recoded the year when the patient was first admitted in our hospital. Finally we obtained data with valid pathological diagnosis in 6,406 cases in the six year period. The methods of this work were described previously^10^,^19^. Among these cases, we obtained 1,973 malignant cases and clinical data were available for 1,710 cases. Clinical data included how the nodule was found, symptoms related to the nodule and the signs during physical examination. We divided the reasons for thyroid nodules consultant into four types: nodule(s) found by the patient, nodule(s) found during routine physical examination, nodule(s) found during consultant for other medical conditions, and for the completion of thyroidectomy after a cancerous nodule resected in other hospitals. Symptoms included discomfort or pain of the front neck, dysphagia, dysphonia or hoarseness and dyspnea. If any of these symptoms occurred, we recorded as positive, otherwise negative. If the nodule(s) or any enlargement of the thyroid gland or local lymph node was palpitated during physical examination, we recorded as positive, otherwise negative.

Generally, thyroidectomies were performed for the following reasons as previously described^19^: a) worrisome findings from ultrasonography, and/or abnormal lymph node enlargement, b) malignancy suspected from previous thyroid FNA, or inclusive FNA results, c) patients with multiple or bilateral nodules or symptoms of neck or throat compression, or enlargement during follow-up, d) concerning clinical or physical examination findings warranting consideration for removal. A small minority of patients chose thyroidectomy at their own wish, primarily for cosmetic reasons.

Malignancy, if present, was reported as one of the following subtypes: papillary thyroid carcinoma (PTC) including crPTC (clinical relevant papillary thyroid carcinoma, with the maximum diameter larger than 10 mm) and microPTC (mPTC) with the maximum diameter less than or equal to 10 mm; follicular thyroid carcinoma (FTC); medullary thyroid carcinoma (MTC); and anaplastic thyroid carcinoma (ATC). For each case, we collected data confirming the maximum diameter of the tumor and other pathologic features as well. All pathological findings were confirmed by experienced endocrine pathologist (Z.Z.). We divided results according to the chronological sequence from 2008 to 2013.

Lymph node dissection was performed at the discretion of the surgeon based upon clinical and other factors. Most often, a neck ultrasound or computed tomography (CT) scan was performed prior to surgery allowing assessment of neck adenopathy. Frozen sections were routinely used to guide the extent of the surgical procedures. If the nodule was found to be malignant by frozen section and no abnormal lymph nodes were identified on preoperative imaging (or during the surgery), an ipsilateral central lymph node dissection (CLND) from level VI was generally performed. Only when the tumor size was within 10 mm and no abnormal lymph node was found during the operation, CLND was not routinely performed. If abnormal lymph nodes were identified during pre-operative ultrasound or intraoperatively, and the snap-frozen sample showed malignant lesion, a modified lateral lymph node dissection (LLND) was performed. All the samples were formalin fixed paraffin embedded (FFPE) for final histopathology confirmation.

Fasting serum-free thyroxine (FT4, reference interval: 12.0–22.0 pmol/l), serum-free triiodothyronine (FT3, reference interval: 3.10–6.80 pmol/l), serum thyroid-stimulating hormone (TSH, reference interval: 0.27–4.2 mIU/L), anti-thyroglobulin (anti-Tg or TgAb, reference interval: 0–115 IU/ml), and anti-thyroid peroxidase (anti-TPO or TPoAb, reference interval: 0–34 IU/ml) were measured by chemiluminescent immunoassay (AutoBio Co., Ltd., Zhengzhou, China) before surgery. Coefficients of variation for the assays were all <15%. Serum TPoAb and TgAb were defined as positive and negative if the value of the antibody was higher than the upper reference or lower than the upper reference, respectively.

Quantitative data were shown as mean ± SD or mean ± SE as indicated, compared using One Way ANOVA whereas numbers and percentage were provided for qualitative data. Percentages were compared using the **χ**^**2**^test or **χ**^**2**^test for trend. Logistic regression test was used to determine the risk factors for local LNM. All analyses were additionally corrected for confounders including age, gender, nodule size, year group and presence of Hashimoto thyroiditis. All tests were 2-sided, and a *P* value < 0.05 was considered statistically significant. Statistical analyses were performed with SPSS software, version 13.0 for Windows (SPSS Inc, Chicago, IL, USA).

### Ethics

This study was reviewed and deemed exempt from written informed consent by the Institutional Review Board (IRB) of the First Affiliated Hospital of Nanjing Medical University. The patient records were anonymized and de-identified prior to analysis. It was approved by the IRB for analysis.

## Results

### Malignancy detection rate from 2008 to 2013

It was found that the rate of malignancy in all thyroid surgeries increased significantly from 2009 to 2013. The malignancy rate of all thyroidectomies increased from 20.6% in 2009 to 41.5% in 2013 significantly (*P* < 0.05, Table 1). In the meantime the benign rate dropped from 79.4% to 58.5% correspondently (*P* < 0.05, Table 1).

### Pathological diagnosis distribution in all thyroid malignancies from 2008 to 2013

In all 1,973 patients of thyroid malignancies, the percentage of papillary thyroid carcinoma (PTC) has significantly increased from 68.5% in 2008 to 93.4% in 2013 (*P* < 0.05, Table 2), while other cancer types decreased or remained similar in detection percentage (Table 2). Majority of the patients had unilateral cancer (74.3%) and only 20.7% of the patients had cancer on both lobes of the thyroid gland. The mean numbers of loci were 1.4 ± 0.8 (Table 2)

### Clinical information for thyroid cancer from 2009 to 2013

**Figure 1:**
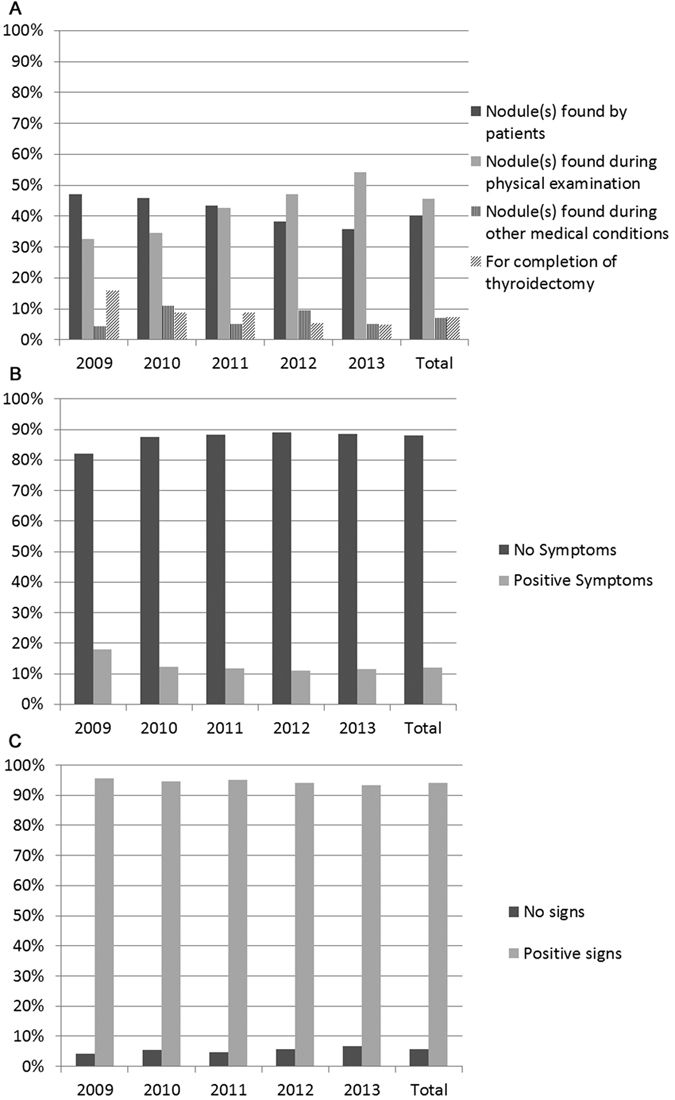
Clinical data related to the patients who received thyroidectomy with malignant histology from 2009 to 2013. (**A**) Reasons for thyroid check-up, (**B**) Symptoms of the patients, (**C**) Signs during physical examination.

Clinical data were obtained for 1,710 cases mostly since 2009. According to the history of each patient in our EMR, we also documented the clinical related information. We found that more patients found their thyroid nodule during routine physical examinations with only 32% in 2009 increased to 54.3% significantly in 2013 (P < 0.05, Fig. 1 and Supplementary Table 1). In the meantime, the percentage of patients who found the nodules by themselves decreased from 47.1% in 2009 to 35.7% in 2013. The patients with the purpose of receiving completion of thyroidectomy also decrease in this period. About 7% of patients found the nodule issue during check-up for other medical conditions which would be termed as incidental thyroid carcinoma (Fig. 1 and Supplementary Table 1).

**Figure 2:**
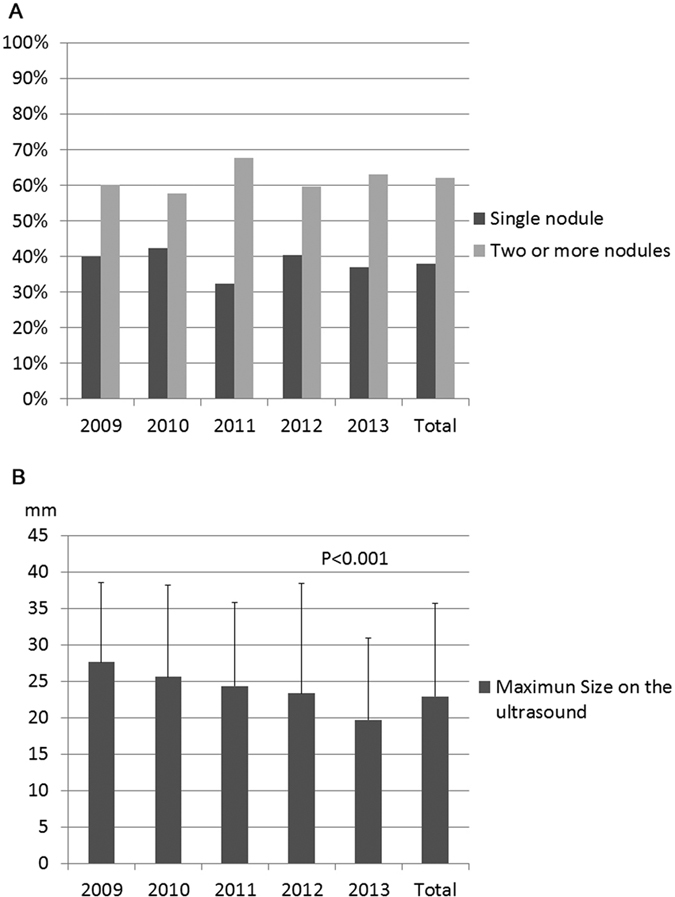
Ultrasound features of the nodules of the patients who had thyroidectomy with malignant histology from 2009 to 2013. (**A**) Multi-nodularity of the patients. (**B**) Mean maximum size of the largest nodule during ultrasonography, data were express as mean ± SD.

**Figure 3:**
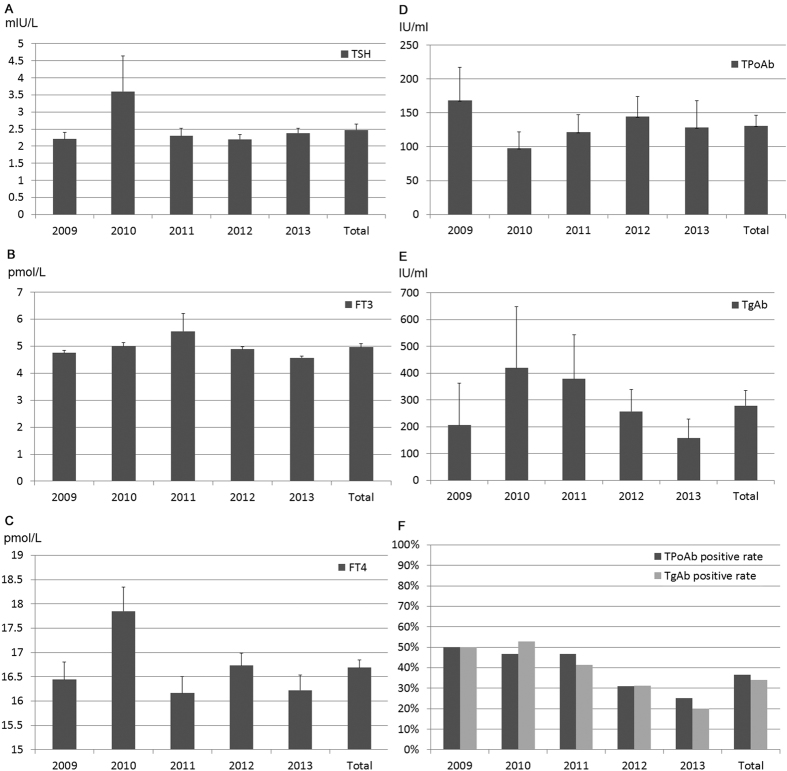
Thyroid function and autoimmune status of the patients who received thyroidectomy with malignant histology from 2009 to 2013. (**A**) Mean levels of TSH (normal reference range 0.27–4.20 mIU/L). (**B**) Mean levels of FT3 (normal reference range 3.10–6.80 pmol/L). (**C**) Mean levels of FT4 (normal reference range 12.00–22.00 pmol/L). (**D**) Mean levels of TPoAb (normal reference range <34.0 IU/ml). (**E**) Mean levels of TgAb (normal reference range <115.0 IU/ml). (**F**) Positivity rates of TPoAb and TgAb. Data were express as mean ± SE.

**Figure 4:**
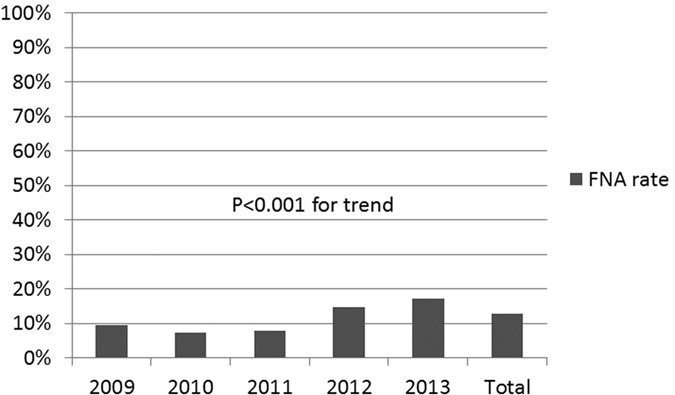
Preoperative FNA rates among the patients who received thyroidectomy with malignant histology from 2009 to 2013. P < 0.001 for trend from 2009 to 2013.

Majority of the patients did not have any clinical related symptoms, only 12% of the patients have different degrees of discomfort including pain and dysphagia etc. (Fig. 1 and Supplementary Table 1). Most of the patients had positive findings including any nodule(s) or type of enlargement of thyroid gland during palpitation of the neck, however 5.8% of the patients didn’t have any positive signs during physical examinations probably due to intra-thyroidal micro-carcinoma in the deep thyroid (Fig. 1 and Supplementary Table 1). During ultrasonography, 38% of the patients had single thyroid nodule and 62% had two or more thyroid nodules. However, the mean maximum size of the thyroid nodule(s) from each patient decreased from 27.6 ± 10.9 in 2009 to 19.7 ± 11.2 in 2013 significantly (Fig. 2 and Supplementary Table 1). The mean levels of thyroid function of these patients were within normal range (Fig. 3 and Supplementary Table 1). The mean levels of thyroid autoantibodies were above normal range, however, the positivity rates for TPoAb and TgAb were all decreased from 2009 to 2013 significantly (Fig. 3 and Supplementary Table 1). The rate of preoperative FNA was all below 10% from 2009 to 2011, however increased gradually from 14.7% in 2012 to 17.2% in 2013 (Fig. 4 and Supplementary Table 1).

### Characteristics of papillary thyroid carcinoma changes from 2008 to 2013

Due to the dominance of PTC, we analysed the detailed features of PTC. The mean age of all the PTC patients was 44.0 ± 13.6 years old, 79.8% being female (Table 3). It was documented that the mean size of PTC dropped significantly from 17.8 ± 14.3 mm in 2008 to 12.2 ± 9.5 mm in 2013 (*P* < 0.05, Table 3). We divided all the PTC into two groups mPTC and crPTC. It was found that the percentage of mPTC was increased from 43.1% in 2008 to 56.5% in 2013 significantly (*P* < 0.05, Table 3). With mPTC being the dominant form of PTC in 2013, the other features including mean age, gender distribution and percentage of LNM remained without significant changes in the past six years annually or in total (*P* > 0.05, Table 3).

In further analysis, we compared the positive lymph node metastasis (LNM) rate between mPTC and crPTC in each year which showed positive LNM was all higher significantly in crPTC group than in mPTC group (Table 4) except that only similar trend was revealed in 2011 although without significance (*P* = 0.055, Table 4). No significance was revealed when comparison of positive LNM percentage was made in consecutive two years or in total six years both in mPTC and in crPTC groups. In general, evidenced positive LNM in all PTC, mPTC and crPTC was 31.3% (Table 3), 20.6% and 41.2% respectively (Table 4) without clear upward or downward trend in the past six years.

### Determinants of local lymph node metastasis in PTC, mPTC and crPTC

**Figure 5:**
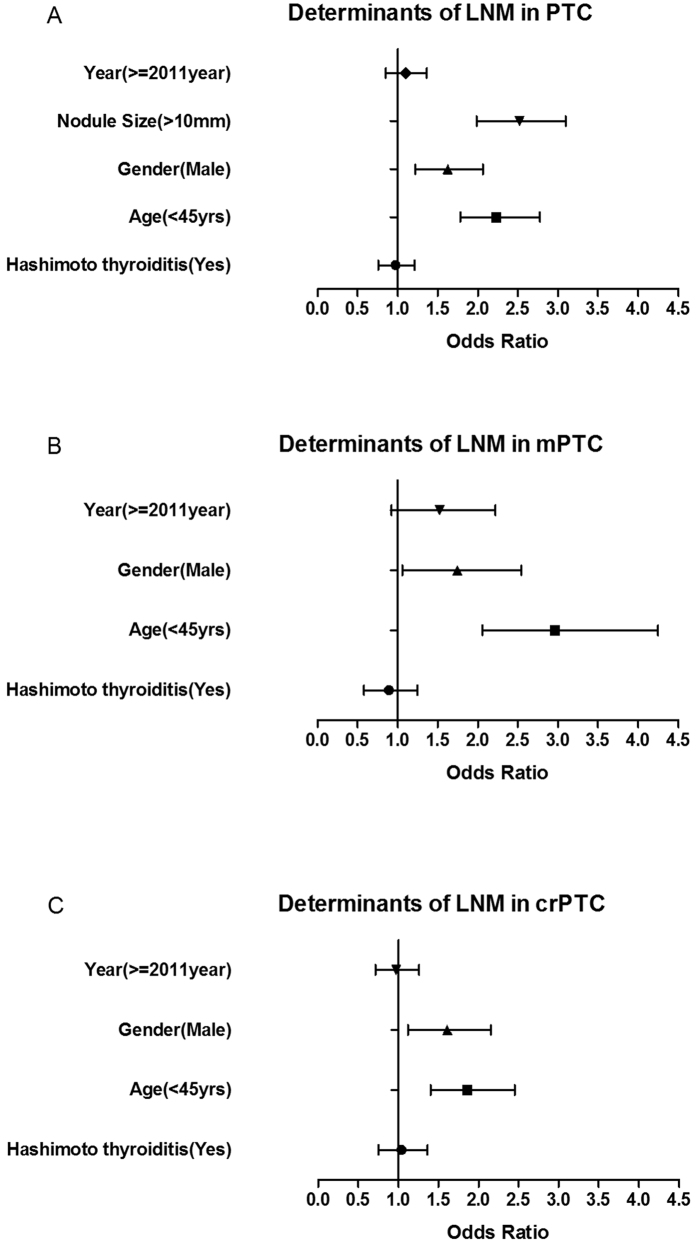
Determinants of Lymph Node Metastasis in PTC, mPTC and crPTC. Logistic regression was used to determine the factors for local lymph node metastasis (LNM) in PTC, mPTC and crPTC after adjustments for other covariates.

We also determined the risk factors for local LNM in PTC, mPTC and crPTC. As showed in Fig. 5 and Supplementary Table 2 the LNM was positively related to nodule size >10 mm (OR = 2.480, CI 1.985–3.098, P < 0.001), male (OR = 1.587, CI 1.220–2.064, P = 0.001) and age below 45 years old (OR = 2.226, CI 1.786–2.774, P < 0.001) after adjustment for other covariates in all PTCs. Consistent findings were revealed in mPTC and crPTC groups (Fig. 5 and Supplementary Tables 3 and 4). However, the year and presence of Hashimoto’s thyroiditis were not related to LNM (Fig. 5 and Supplementary 2–4).

## Discussion

Our study reported significantly increased malignancy detection rate in the past six years reaching 41.5% in 2013 which was favorably higher than 28.1% in Malaysia^4^ and 6.7% in Germany^20^. However, this figure was still lower than 56% in a study from Brigham and Women’s Hospital (BWH), one of the major affiliates of Harvard Medical School in the United States^21^. The discrepancy behind this might be related to different algorithms physicians or surgeons were using for patients with thyroid nodules reflecting different background of medical insurance and cost. Using ultrasound as one single tool might not be accurate enough for preoperative diagnosis^22^. Combination of FNA, standardized cytology reporting system, repeated biopsy for undetermined thyroid nodules and molecular diagnosis may provide more precise preoperative diagnosis and result in a higher malignancy detection rate^9^,^11^,^23^,^24^,^25^,^26^,^27^,^28^,^29^,^30^,^31^,^32^,^33^,^34^,^35^,^36^,^37^. One important factor might be low FNA rate in our cohort. We only had 12.8% of the patients who received FNA preoperatively from 2009 to 2013, however, at BWH 100% of patients received FNA before surgery^21^. Similar situation was also found in Germany which also resulted a much lower malignancy rate^20^. We also found that preoperative FNA was significantly increased during these year which correlated to the increased cancer detection rate implying the FNA remains the main step when diagnosing thyroid nodule(s) as suggested by ATA guidelines^5^,^23^. It is reasonable to believe a multi-disciplinary approach including a routine preoperative FNA might be needed to achieve higher malignancy detection rate in the future^21^,^28^.

With higher malignancy detection rate, the major form of malignancy remained to be PTC reaching 93.4% in 2013 and 86.4% averagely in the six-year period which were both higher than previous reports^38^. This significantly increased distribution of PTC in all thyroid cancer might be due to patient selection bias and the more recognition of high risk appearances for PTC under the ultrasound. Several ultrasonographic features have been shown to be related to thyroid malignancy, for example, marked hypoechogenicity, irregular or microlobulated margins, a taller than wide configuration (anteroposterior dimension greater than transverse dimension), microcalcification, or chaotic arrangement of intranodular vascular images and presence of metastatic lymph nodes or extracapsular growth^5^. Among them, microcalcification is believed to have a high specificity for PTC^12^,^23^. The question rises whether FNA or surgery is needed for when highly suspicious ultrasound features are reported in a sub-centimeter lesion. According the recent guideline from ATA, these sub-centimeter nodules might not need FNA in the absence of highly suspicious LN based on the fact that vast majority of these microPTC (if confirmed) either progress very slow or not at all^39^. However, according to the data from the current study, this was not entirely true. We did found that around 20% of all these micro-PTC already showed the positive local lymph node metastasis. If we set the FNA cut-off as nodules with maximum diameter larger than 1 cm as recommended by ATA, all these microPTCs with LNM could not have been confirmed histologically. On the other hand, for the rest 80% of the micro-PTCs, the ATA guideline seemed to guide the right way at least from the perspective of LNM. The question would lie in more about when we need to be more aggressive and when to me more conservative for those highly suspicious nodule within 1 cm. Our analysis suggests that for male patients with younger age (<45 years old), more attention may be needed to be paid as the risk of LNM in this group of patients is high. There has been a major area of controversy that whether these sub-centimeter PTCs needed to be treated as crPTC^40^,^41^. Some investigators advocated in favor of not performing further treatment in addition to initial thyroid surgery and some even suggest to remove the cancer label from indolent lesions^2^,^42^, whereas others suggested an aggressive surgical approach followed by radioiodine ablation therapy^40^. Dr Miyauchi compared the outcome of immediate surgery and active surveillance for patients with microPTC^43^. The oncological outcomes were similarly excellent, but the incidences of unfavorable events were definitely higher in the immediate surgery group. He has suggested that active surveillance is now recommended as the best choice for patients with low-risk mPTC. A recent publication^44^ suggested specific recommendations with regard to the optimal selection of patients with microPTCs for an active surveillance. They described a risk-stratified clinical decision-making framework^44^. This framework was composed with three major factors, tumor/neck US characteristics, patient characteristics and medical team characteristics^44^. They have described as inappropriate to observe or follow-up the patients if there is evidence of aggressive cytology on FNA (rare), or subcapsular locations adjacent to recurrent laryngeal nerve (RLN), or evidence of extrathyroidal extension or clinical evidence of invasion of RLN or trachea (rare) or N1 disease at initial evaluation, etc. Although in our data we have not been able to show all the detailed information described above, they do mention the LNM as a factor to include the patient as the candidate for surgery and FNA should be done for these sub-centimeter lesions to exclude aggressive form of thyroid cancer. We found this framework would be more reasonable to apply when facing individual patient.

The main limitation of current study was that all the data were from a single academic center with a retrospective nature. Patient selection bias was unavoidable. We only compared the local LNM, other pathological features such as extrathyroidal extension, capsule invasion were not compared which are also recognized as important prognostic information^45^. There were a certain percentage of lymph nodes not resected during the thyroidectomies. We did not have robust follow-up evidence, as suggested by many studies that PTC as the major form of thyroid cancer is generally indolent in nature and longer time of follow-up is often suggested^46^,^47^,^48^,^49^. For a future study, we hope to analyze the follow up data as to further confirm our points.

Our study has filled in the blanks of thyroid research by showing the evolutionary trend of thyroid carcinoma from a single academic center in China. The major findings of the current study lie in that mPTC has become the dominant form of thyroid cancer without significant change in local LNM in recent years. Although the detection rate of mPTC was indeed increased from 43.1% to 56.5%, the local lymph node metastasis was generally around 20% suggesting than around 80% of these microPTCs did not have evidence of cancer progression as manifested by LNM. Our findings support this hypothesis that an increasing number of thyroidectomies are being performed for small and often indolent disease questioning the current algorithms surgeons are using for patients with thyroid nodules in China. Our study provided some evidence for physicians or surgeons that clinical judgments are very important independent of the nodule size especially when high risk ultrasound appearances or suspicious LN were present at first clinical consult. A risk-stratified clinical decision-making framework might be useful when making decisions for these sub-centimeter lesions especially when microPTC is confirmed by FNA, however further study is needed to illustrate this issue.

## Additional Information

**How to cite this article**: Liu, X. *et al*. Evolutionary features of thyroid cancer in patients with thyroidectomies from 2008 to 2013 in China. *Sci. Rep.* **6**, 28414; doi: 10.1038/srep28414 (2016).

## Supplementary Material

Supplementary Information

## Figures and Tables

**Table 1:** Malignancy and benignity percentage detected during thyroidectomies from 2008 to 2013.

	**2008**	**2009**	**2010**	**2011**	**2012**	**2013**	**Total**	**P**
Benign, n(%)	612(75.4%)	742(79.4%^a^)	746(73.5%^a^)	765(70.5%)	737(64.6%^a^)	831(58.5%^a,b^)	4433(69.2%)	<0.001
Cancer, n(%)	200(24.6%)	192(20.6%^a^)	269(26.5%^a^)	320(29.5%)	403(35.4%^a^)	589(41.5%^a,b^)	1973(30.8%)	
Total	812	934	1015	1085	1140	1420	6406	

^a^means percentages were significantly different compared with previous year; ^b^means percentages were significantly different compared with the year 2008.

**Table 2:** Pathological diagnosis classification of all thyroid malignancies from 2008 to 2013.

	**2008**	**2009**	**2010**	**2011**	**2012**	**2013**	**Total**	**P for difference**	**P for trend**
PTC, n(%)	137(68.5%)	146(76.0%^a^)	230(85.5%^a^)	277(86.6%)	364(90.3%)	550(93.4%^b^)	1704(86.4%)	<0.001	<0.001
FTC, n(%)	42(21.0%)	13(6.8%^a^)	12(4.5%)	15(4.7%)	13(3.2%)	12(2.0%^b^)	107(5.4%)	<0.001	<0.001
MTC, n(%)	6(3.0%)	2(1.0%)	5(1.9%)	6(1.9%)	9(2.2%)	7(1.2%)	35(1.8%)	0.550	0.318
ATC, n(%)	0(0.0%)	1(0.5%)	2(0.7%)	0(0.0%)	1(0.2%)	0(0.0%)	4(0.2%)	0.210	0.274
Other, n(%)	15(7.3%)	30(15.6%^a^)	20(7.4%^a^)	22(6.9%)	16(4.0%)	20(3.4%)	123(6.2%)	<0.001	<0.001
Unilateral or Bilateral									
Unilateral	141(70.5%)	131(68.2%)	204(75.8%)	234(73.1%)	299(74.2%)	456(77.4%)	1465(74.3%)	0.118	0.016
Bilateral	45(22.5%)	35(18.2%)	49(18.2%)	67(20.9%)	94(23.3%)	119(20.2%)	409(20.7%)	0.563	0.740
Unknown*	14(7%)	26(13.5%)	16(5.9%)	19(5.9%)	10(2.5%)	14(2.4%)	99(5.0%)	<0.001	<0.001
Mean numbers of loci (n)**	1.45 ± 0.76	1.46 ± 0.85	1.46 ± 0.91	1.41 ± 0.73	1.49 ± 0.83	1.42 ± 0.79	1.44 ± 0.81	0.826	
All cancer(n)	200	192	269	320	403	589	1973		

^a^means percentages were significantly different compared with previous year; ^b^means percentages were significantly different compared with the year 2008; PTC, papillary thyroid carcinoma; FTC, follicular thyroid carcinoma; MTC, medullary thyroid carcinoma; ATC, anaplastic thyroid carcinoma; other means all the carcinoma could not be classified into previous types including poorly differentiated thyroid cancer, squamous cell carcinomas, B cell lymphomas of thyroid, spindle cell carcinoma, adenoid cystic carcinoma, renal clear cell metastasis and Langerhans cell histiocytosis of the thyroid gland.

^*^Probably due to primary surgeries were done at other hosptials.

^**^data were presented as mean ± SD.

**Table 3:** Characteristics of PTCs from 2008 to 2013.

	**2008**	**2009**	**2010**	**2011**	**2012**	**2013**	**Total**	**P for difference**	**P for trend**
Age(year)	43.5 ± 14.8	43.5 ± 13.9	45.9 ± 14.7	44.5 ± 13.7	43.5 ± 13.1	43.5 ± 13.1	44.0 ± 13.6		
Male, n(%)	29(21.2%)	31(21.2%)	42(18.3%)	49(17.7%)	87(23.9%)	106(19.3%)	344(20.2%)	0.394	0.976
Female, n(%)	108(78.8%)	115(78.8%)	188(81.7%)	228(82.3%)	277(76.1%)	444(80.7%)	1360(79.8%)		
Mean Diameter(mm)*	17.8 ± 14.3	16.8 ± 11.9	16.6 ± 12.2	15.4 ± 11.2	14.4 ± 11.8	12.2 ± 9.5^a,b^	14.6 ± 11.5	<0.001	
crPTC, n(%)	78(56.9%)	94(64.4%)	141(61.3%)	154(55.6%)	183(50.3%)	239(43.5%^a,b^)	889(52.2%)	<0.001	<0.001
mPTC, n(%)	59(43.1%)	52(35.6%)	89(38.7%)	123(44.4%)	181(49.7%)	311(56.5%^a,b^)	815(47.8%)	<0.001	<0.001
Postive LNM, n(%)	37(27.0%)	51(34.9%)	73(31.7%)	84(30.3%)	122(33.5%)	167(30.4%)	534(31.3%)	0.659	0.907
Negative LNM, n(%)	32(23.4%)	37(25.3%)	49(21.3%)	48(17.3%)	80(22.0%)	134(24.4%)	380(22.3%)	0.276	0.703
No LN Resected, n(%)	68(49.6%)	58(39.7%)	108(47.0%)	145(52.3%)	162(44.5%)	249(45.3%)	790(46.4%)	0.158	0.670
Total	137	146	230	277	364	550	1704		

^a^means percentages were significantly different compared with previous year; ^b^means percentages were significantly different compared with the year 2008; crPTC, clinical relevant papillary thyroid carcinoma; mPTC, micro papillary thyroid carcinoma; LNM, lymph node metastasis; LN, lymph node.

^*^Data were presented as mean ± SD.

**Table 4:** Rate of local lymph node metastasis comparison between mPTC and crPTC from 2008 to 2013.

	**2008**	**2009**	**2010**	**2011**	**2012**	**2013**	**Total**	**P for difference**	**P for the trend**
Positive LNM in mPTC, n(%)	9(15.3%)	8(15.4%)	14(15.7%)	30(24.4%)	40(22.1%)	67(21.5%)	168(20.6%)	0.456	0.128
Positive LNM in crPTC, n(%)	28(35.9%)	43(45.7%)	59(41.8%)	54(35.1%)	82(44.8%)	100(41.8%)	366(41.2%)	0.398	0.596
P value*	0.007	<0.001	<0.001	0.055	<0.001	<0.001	<0.001		

^*^Rate of Positive LNM was compared between mPTC and crPTC in each year. LNM, lymph node metastasis; mPTC, micro papillary thyroid carcinoma; crPTC, clinical relevant papillary thyroid carcinoma.
