# Supplementary material for: Evolutionary features of thyroid cancer in patients with thyroidectomies from 2008 to 2013 in China
Source: Sci Rep. 2016 Jun 22;6:28414. doi: 10.1038/srep28414 (PMC4916471; doi:10.1038/srep28414)
Supplement: Supplementary Information [file srep28414-s1.doc]

**Title Page**

**Evolutionary features of thyroid cancer in patients with thyroidectomies from 2008 to 2013 in China**

Running title: Evolutionary features of thyroid cancer

**Xiaoyun Liu^1^*, Lijun Zhu^2^*, Zhixiao Wang^1^, Dai Cui^1^, Huanhuan Chen^1^, Yu Duan^1^, Meiping Shen^3^, Hui Lu^3^, Zhihong Zhang^4^, Jiawei Chen^1^, Erik K. Alexander^5^, Tao Yang^1#^ and Xiaodong Wang^1#^**

*^1^Department of Endocrinology, The First Affiliated Hospital of Nanjing Medical University, Nanjing, China, ^2^Department of Children’s Health care, Nanjing Maternity and Child Health Care Hospital Affiliated to Nanjing Medical University, China, ^3^Department of Surgery, The First Affiliated Hospital of Nanjing Medical University, Nanjing, China, ^4^Department of Pathology, The First Affiliated Hospital of Nanjing Medical University, Nanjing, China, ^5^Thyroid Unit, Division of Endocrinology, Metabolism and Diabetes, Department of Medicine, Brigham & Women’s Hospital and Harvard Medical School, Boston, USA (*X.L. and L.Z. contributed equally to this work)*

**Corresponding author:**

**#Tao Yang, MD, PhD**

**Department of Endocrinology, The First Affiliated Hospital of Nanjing Medical University, Nanjing, China 210029, +86 13851498409 (Phone), E-mail:** [**yangt@njmu.edu.cn**](mailto:yangt@njmu.edu.cn)

**and**

**#Xiaodong Wang, MD**

**Department of Endocrinology, The First Affiliated Hospital of Nanjing Medical University, Nanjing, China 210029, +86 13705176611(Phone), E-mail:** [**drwangxd@vip.163.com**](mailto:drwangxd@vip.163.com)

Supplementary Table 1. Clinical findings for thyroidectomies with malignant histology results from 2009-2013

|  | 2009 | 2010 | 2011 | 2012 | 2013 | Total | P for difference | P for trend |
| --- | --- | --- | --- | --- | --- | --- | --- | --- |
| Reasons for thyroid check-up |  |  |  | | | | | |
| Nodule(s) found by patients | 47.1% | 45.8% | 43.5% | 38.2% | 35.7% | 40.2% | 0.010 | <0.001 |
| Nodule(s) found by physical examination | 32.6% | 34.5% ^a^ | 42.6% ^a^ | 47.1% ^a^ | 54.3%^a,b^ | 45.6% | <0.001 | <0.001 |
| Nodule(s) found during other medical conditions | 4.3% | 11.0% | 5.0% | 9.4% | 5.1% | 7.0% | 0.002 | 0.267 |
| For completion of thyroidectomy | 15.9% | 8.7% | 8.8% | 5.2% | 4.9% | 7.2% | <0.001 | <0.001 |
| Symptoms |  |  |  |  |  |  |  |  |
| No Symptoms | 82.1% | 87.6% | 88.3% | 89.0% | 88.5% | 88.0% | 0.343 | 0.139 |
| Positive Symptoms | 17.9% | 12.4% | 11.7% | 11. 0% | 11.5% | 12.0% |  |  |
| Signs |  |  |  |  |  |  |  |  |
| No signs | 4.3% | 5.4% | 4.8% | 5.8% | 6.7% | 5.8% | 0.734 | 0.204 |
| Positive signs | 95.7% | 94.6% | 95.2% | 94.2% | 93.3% | 94.2% |  |  |
| Ultrasound findings |  |  |  |  |  |  |  |  |
| Single nodule | 39.8% | 42.3% | 32.3% | 40.4% | 37.0% | 38.0% | 0.132 | 0.526 |
| Two or more nodules | 60.2% | 57.7% | 67.7% | 59.6% | 63.0% | 62.0% |  |  |
| Maximun Size on the ultrasound (mm) | 27.63±10.92 | 25.63±12.62 | 24.33±11.51 | 23.35±15.14 | 19.72±11.18^a,b^ | 22.92±12.78 | <0.001 |  |
| Thyroid function and thyroid antibodies |  |  |  | | | | | |
| TSH mIU/L (0.27-4.20)* | 2.21±0.19 | 3.60±1.04^a^ | 2.30±0.23^a^ | 2.2±0.14 | 2.38±0.15 | 2.47±0.18 | 0.120 |  |
| FT3 pmol/L (3.10-6.80) * | 4.75±0.10 | 5.00±0.14 | 5.55±0.66 | 4.89±0.08 | 4.57±0.07 | 4.96±0.13 | 0.218 |  |
| FT4 pmol/L (12.00-22.00) * | 16.44±0.36 | 17.85±0.50^a^ | 16.16±0.34^a^ | 16.73±0.25 | 16.22±0.31 | 16.69±0.16 | 0.011 |  |
| TPoAb IU/ml (<34.0) * | 168.43±48.45 | 97.30±24.42 | 121.31±25.72 | 144.29±29.69 | 128.02±39.77 | 130.78±15.64 | 0.823 |  |
| TPoAb positive rate | 50.00% | 46.80% | 46.8% | 30.9% | 25.00%^b^ | 36.5% | 0.038 | 0.003 |
| TgAb IU/mL (<115.0*) | 205.70±156.51 | 419.46±229.21 | 378.37±165.34 | 257.45±81.06 | 156.74±72.52 | 278.51±57.87 | 0.682 |  |
| TgAb positive rate | 50.00% | 52.90% | 41.4% | 31.3% | 20.00% | 34.00% | 0.152 | 0.011 |
| FNA rate | 9.4% | 7.4% | 7.9% | 14.7%^a^ | 17.2%^b^ | 12.8% | <0.001 | <0.001 |

a indicates percentages were significantly different compared with previous year

b indicates percentages were significantly different compared with the year 2009

*normal reference range, data were presented as mean±SE

Supplementary Table 2. Determinants of Lymph node metastasis in PTC

| Independent variable | Univariate | | Multivariate | |
| --- | --- | --- | --- | --- |
|  | OR(95% CI) | *P* value | OR(95% CI) | *P* value |
| Hashimoto thyroiditis(Yes) | 0.948(0.771-1.190) | 0.699 | 0.958(0.758-1.212) | 0.722 |
| Age(<45yrs) | 2.406(1.945-2.976) | <0.001 | 2.226(1.786-2.774) | <0.001 |
| Gender(Male) | 1.709(1.338-2.183) | <0.001 | 1.587(1.220-2.064) | 0.001 |
| Nodule Size(>10mm) | 2.695(2.172-3.345) | <0.001 | 2.480(1.985-3.098) | <0.001 |
| Year(>=2011year) | 0.997(0.798-1.246) | 0.979 | 1.079(0.853-1.366) | 0.526 |

Supplementary Table 3. Determinants of Lymph node metastasis in mPTC

| Independent variable | Univariate | | Multivariate | |
| --- | --- | --- | --- | --- |
|  | OR(95% CI) | *P* value | OR(95% CI) | *P* value |
| Hashimoto thyroiditis(Yes) | 0.944(0.659-1.352) | 0.754 | 0.850(0.579-1.248) | 0.407 |
| Age(<45yrs) | 2.973(2.084-4.240) | <0.001 | 2.959(2.060-4.252) | <0.001 |
| Gender(Male) | 1.730(1.143-2.618) | 0.010 | 1.639(1.057-2.543) | 0.027 |
| Year(>=2011year) | 1.562(1.019-2.396) | 0.041 | 1.430(0.922-2.218) | 0.110 |

Supplementary Table 4. Determinants of Lymph node metastasis in crPTC

| Independent variable | Univariate | | Multivariate | |
| --- | --- | --- | --- | --- |
|  | OR(95% CI) | *P* value | OR(95% CI) | *P* value |
| Hashimoto thyroiditis(Yes) | 0.982(0.740-1.304) | 0.901 | 1.011(0.750-1.362) | 1.011 |
| Age(<45yrs) | 1.849(1.403-2.436) | <0.001 | 1.856(1.404-2.452) | <0.001 |
| Gender(Male) | 1.536(1.122-2.102) | 0.007 | 1.553(1.119-2.155) | 0.009 |
| Year(>=2011year) | 0.977(0.739-1.292) | 0.871 | 0.948(0.714-1.259) | 0.712 |
